# Updates on Hepatitis B Virus (HBV) Infection 2.0

**DOI:** 10.3390/microorganisms11122874

**Published:** 2023-11-27

**Authors:** Isabelle Chemin, Flor Helene Pujol

**Affiliations:** 1 Institut National de la Santé et de la Recherche Médicale (Inserm) U1052, Centre de Recherche en Cancérologie de Lyon (CRCL), 151 Cours Albert Thomas, 69003 Lyon, France; 2 Laboratorio de Virología Molecular, Centro de Microbiología y Biología Celular (CMBC), Instituto Venezolano de Investigaciones Científicas (IVIC), Caracas 1020A, Venezuela; 3 Collegium de Lyon, Institut d’Etudes Avancées, Université Lyon 2, 69007 Lyon, France

Hepatitis B is a “silent epidemic” that is fifty to a hundred (50–100) times more infectious than HIV and is a potentially life-threatening liver infection [1]. Hepatitis B virus (HBV) infection can cause acute and chronic disease and subsequently results in a high risk of death from cirrhosis and liver cancer. Despite the availability of a safe and effective vaccine, HBV continues to be a global burden due to mother-to-child transmissions, insufficient HBV vaccinations, and a significant number (290 M) of chronic carriers [2,3]. To achieve the WHO goal of eliminating HBV by 2030, policies targeting infection transmission control among risk groups, community awareness programs, research, drug price reductions, mass vaccinations, and diagnostics should be urgently instituted [4]. HBV exhibits significant genetic variability, primarily attributed to its high mutation rate during viral replication. This genetic variability has important clinical implications. The virus has been classified into different 10 genotypes and numerous sub-genotypes, with some of them less studied than others [5].

Some studies suggest that certain genotypes are linked to more severe liver disease, an increased risk of cirrhosis, and a higher likelihood of developing hepatocellular carcinoma (HCC) [6,7]. The genetic variability of HBV can also influence the response to antiviral therapy. For instance, specific mutations in the viral genome, such as those in the reverse transcriptase region of the polymerase gene, can confer resistance to certain antiviral drugs. Understanding the viral genotype is crucial for tailoring treatment strategies. The genetic diversity of HBV can also pose challenges in diagnostic testing. For example, variations in the viral genome can affect the accuracy of nucleic acid tests used to detect and quantify HBV DNA. This variability needs to be considered when interpreting test results. Different genotypes and sub-genotypes of HBV have distinct geographical distributions. Understanding the prevalent genotypes in a particular region is important for public health planning, as it might impact the design of treatment strategy follow-up programs. Ongoing research may provide additional insights into the clinical impact of hepatitis B variability: the most studied genotype of HBV, genotype D, is also the most widespread [8]. In some settings, healthcare professionals consider the viral genotype when managing patients with chronic hepatitis B to optimize treatment outcomes.

HBV variability can influence its transmission dynamics. Some genotypes may be associated with higher rates of vertical transmission, i.e., from mother to child [9], or horizontal transmission (among adults through blood or sexual contact).

Hepatitis D, also known as delta hepatitis, is a liver infection caused by the hepatitis D virus (HDV). When HDV infects a person already infected with HBV, it can lead to more severe liver disease compared to infection with HBV alone. The interplay between hepatitis B, hepatitis D, inflammation, and oxidative stress can have significant implications for liver health.

Some individuals can be infected with both HBV and HDV simultaneously. Co-infection tends to result in more severe acute hepatitis. Individuals with chronic HBV infection can also be superinfected with HDV. This superinfection often leads to more severe liver disease, including an increased risk of cirrhosis and hepatocellular carcinoma.

The presence of both HBV and HDV can lead to increased inflammation in the liver [10]. Chronic inflammation is a key driver of liver damage and progression to cirrhosis. The immune response to the viruses, especially when they are replicating actively, contributes to this inflammation. Chronic viral infections, including hepatitis B and D, can contribute to oxidative stress in the liver [11]. Oxidative stress results from an imbalance between the production of reactive oxygen species (ROS) and the body’s ability to neutralize them. It can further contribute to liver injury and inflammation. The immune response against HBV and HDV can cause significant tissue damage. This damage, combined with ongoing inflammation and oxidative stress, can lead to the formation of fibrous tissue in the liver (fibrosis) and, eventually, cirrhosis.

This Special Issue explores the viral characteristics of this pathogen, particularly its genetic diversity, together with several aspects of its pathogenicity, including the peculiar interplay of this virus with its satellite virus HDV.
